# Glasgow University and Medical School

**Published:** 1887-01-08

**Authors:** M. Thomas

**Affiliations:** Superintendent. Royal Infirmary, Glasgow


### GLASGOW UNIVERSITY AND MEDICAL SCHOOL.

IN THE HOSPITAL of the 11th inst., you gave the minimum cost which a student may be expected to expend during his three or four years' attendance on classes for the diploma of L.F.P.S. Glasgow, at from £300 to £350, and from £500 to £550 for the M.D. of the University of Glasgow. I understand these sums include the cost of the diploma or the degree. The L.F.P.S.Glas. is now merged in the triple qualification of the Royal Colleges of Physicians and Surgeons, and the Faculty of Physicians and Surgeons. Your estimate is, in my opinion, too high; a student could live luxuriously on the sums you mention. I think a student whose habits are not extravagant, can complete a very full curriculum at this school, and take the triple qualification for a sum of £270; or if he attends the University and takes his M.D., he could do it comfortably for £310. Many hard-working students with limited means do it for considerably less.

M. THOMAS, M.D., Superintendent.
Royal Infirmary, Glasgow.

